# Machine-learning prediction of hosts of novel coronaviruses requires caution as it may affect wildlife conservation

**DOI:** 10.1038/s41467-022-32746-7

**Published:** 2022-09-12

**Authors:** Sophie Lund Rasmussen, Cino Pertoldi, David W. Macdonald

**Affiliations:** 1 Wildlife Conservation Research Unit, The Recanati-Kaplan Centre, Department of Biology, University of Oxford, Tubney House, Abingdon Road, Tubney, Abingdon, OX13 5QL UK; 2 Department of Chemistry and Bioscience, Aalborg University, Fredrik Bajers Vej 7H, DK-9220 Aalborg, Denmark; 3 Aalborg Zoo, Mølleparkvej 63, DK-9000 Aalborg, Denmark

**Keywords:** Machine learning, Viral reservoirs, Viral evolution

**arising from** Wardeh et al. *Nature Communications* 10.1038/s41467-021-21034-5 (2021)

A study published in Nature Communications^1^ used a similarity-based machine-learning pipeline approach to predict associations between coronaviruses and their potential mammalian hosts. Based on this, the authors identified a number of species predicted to have the potential to become recombination hosts for future outbreaks of coronaviruses. If correct, such predictions would have important epizootiological and public health consequences, but if not correct it could nonetheless foster public fears and perceptions in ways that unintentionally undermine wildlife conservation efforts. It is therefore important to be confident in the predictions, which would be weakened by any inadequacies in the data input into the machine-learning and artificial intelligence analyses. Using the case of the European hedgehog (*Erinaceus europaeus*) for illustration, we suggest that such inadequacies do weaken the predictions of the study, and therefore unnecessarily raise public anxieties about proximity to this already threatened species.

Machine-learning algorithms are efficient tools for analysing large datasets. This powerful technology can be applied innovatively to solve a range of challenges with classification and regression of comprehensive datasets or identifying trends and patterns. These algorithms are capable of learning from the data provided, but this powerful capacity inevitably suffers the potential weakness of being misled if the input data attend incompletely to relevant biological factors.

A recent influential paper by Wardeh et al.^1^ uses a similarity-based machine-learning pipeline approach to ask which mammalian species are potential sources for generating new coronaviruses. Without detracting from the importance of this question, we draw attention to the public and policy hazards arising if machine learning suggests biologically unlikely host-pathogen interactions that raise unwarranted fears in an already anxious public—in this case by raising the specter that European hedgehogs are likely to become hosts of novel coronaviruses, thereby risking undermining the already frail conservation status of this declining species.

Many species of wildlife carry host-specific genetically distinct strains of coronaviruses which cannot infect humans. The coronavirus previously detected in hedgehogs is called EriCoV^2–5^. The prevalence of EriCoV in hedgehogs appears to be rather high, ranging from 10.8% in Great Britain^3^ to 50% in France^6^ to 58.3% in Italy^2^, and 58.9% in Germany^4^.

Wardeh, et al.^1^ apply what they call a ‘no-preconceptions’ approach, with the intention of analysing data without being restricted by *“*current incomplete knowledge of the specific biological and molecular mechanisms, which govern host-virus permissibility*”*. Consequently, they deliberately excluded data on e.g. angiotensin I converting enzyme 2 (ACE2) receptors in their computational analyses. However, while omitting such information may avoid some preconceptions, it may also cause the algorithm predicting recombination events to over-represent host-pathogen interactions.

The main receptor of SARS-CoV-2, ACE2 is needed for the virus to infect cells and therefore individuals. We are aware of studies that discuss limitations to the use of ACE2 receptors as predictors of susceptibility to SARS-CoV-2 in wildlife, see for example Delahay et al.^7^ and Martínez-Hernández et al.^8^. However, we know of four articles whose authors conclude that European hedgehogs are unlikely hosts to SARS-CoV-2 due to the extensive genetic differences in their ACE2 receptors compared to the human variant^9–12^. Wu et al.^12^ used several approaches to analyse the ACE2 genes (orthologs) from a range of animal species, including European hedgehogs, and the interactions with the SARS-CoV-2 receptor binding domain (RBD). They experimentally exposed cells containing the hedgehog ACE2 ortholog to a “SARS-CoV pseudovirus”, and did not observe infection, concluding that hedgehogs are not susceptible to SARS-CoV-2. Luan, et al.^11^ performed homology modelling of the SARS‐CoV‐2 spike protein with ACE2 of several mammalian species and predicted that SARS‐CoV‐2 could not bind to the ACE2 receptor of the European hedgehog, as it lacked the five key amino acids critical for binding the spike protein of human SARS-related coronavirus (SARSr-CoV) to the ACE2 receptor, which is a prerequisite for SARS-CoV-2 to enter and infect a cell.

These articles^9–12^ all conclude that European hedgehogs appear not to be susceptible to SARS-CoV-2, a conclusion in line with the fact that SARS-CoV-2 has never been detected in this species, despite the opportunity for emerging mutations altering the virus.

Wardeh et al.^1^ state: “Our results also implicate the common hedgehog (*Erinaceus europaeus*), the European rabbit (*Oryctolagus cuniculus*), and the domestic cat (*Felis catus*) as predicted hosts for SARS-CoV-2 (confirmed for the cat) …Our prediction of these species’ potential interaction with SARS-CoV-2 and considerable numbers of other coronaviruses, as well as the latter three species’ close association to humans, identify them as high priority underestimated risks”.

Three considerations have bearing on this conclusion:

Firstly, the authors record that SARS-CoV-2 has not been detected in hedgehogs and no other hosts have yet been identified for EriCoV (their Supplementary Data 1, 2 in ref. 1), and yet, the algorithm predicted a probability of 0.78 for EriCoV to infect humans (Supplementary Data 5 in ref. 1). It is not clear, in the absence of any cases of EriCoV in humans, how this co-infection could occur, unless considering another scenario where the co-infection with EriCoV and SARS-CoV-2, or another wildlife strain of coronavirus, would occur in another wildlife host and cause recombination.

Secondly, the authors state that the algorithm reveals that “considerable numbers” of other coronaviruses could potentially recombine with the EriCoV carried by hedgehogs. Although from the data provided in the article and supplementary files we have been unable to discern which other coronaviruses and hosts they refer to, it appears that three to four coronavirus genera (Supplementary Data 4 in ref. 1) and up to 21 coronaviruses (Supplementary Data 3 in ref. 1) are predicted to be candidates for recombination in hedgehogs with the EriCoV carried by hedgehogs, or with the co-infection with EriCoV happening in another species. The obvious naturalistic question is whether these potentially co-infectious coronavirus strains have been detected in species with which the hedgehogs are sympatric, or likely to interact. This question may have been partly addressed by the inclusion in the analyses of habitat type, but only insofar as the algorithm took account of whether these habitats occurred within overlapping zones of the geographical ranges of each species.

Thirdly, while we agree that the sometimes close associations between hedgehogs and humans could provide a theoretical risk of zoonotic transfers (for hedgehog rehabilitators, hedgehog researchers and those provisioning hedgehogs in their gardens), De Sabato et al.^5^ estimate that EriCoV originated between 190 and 1447 years ago, and in spite of its long history and high prevalence in hedgehogs, has not yet been recorded to have infected any other host, including humans. Furthermore, our research on methicillin-resistant *Staphylococcus aureus* (MRSA) indicates that the risk of zoonotic transfers between humans and hedgehogs appears, in practice, to be low^13^.

Wardeh et al.^1^ argue that one of the greatest risks from new variants of coronaviruses lies in co-infections with SARS-CoV-2 and another coronavirus potentially leading to homologous recombination. In the case of the hedgehog, this would necessitate the simultaneous infection in hedgehogs or humans (or a third host) with SARS-CoV-2 (there is no evidence that it can infect hedgehogs), and EriCoV (which belongs to a different genetic clade than SARS-CoV-2 and to which there is no evidence that humans are susceptible) - a seemingly unlikely circumstance given the substantial genetic differences between humans and hedgehogs at key points of the molecular structures required for SARS-CoV-2 to infect. Furthermore, Corman, et al.^4^ suggest that the EriCoV has a low potential to replicate in heterologous host cells, raising the possibility that EriCoV is so host-specific as to be incapable of infecting other species at present. However, as humans can become infected with other variants of merbecovirus, such as Middle East respiratory syndrome coronavirus (MERS-CoV) one cannot rule out a theoretical risk of EriCoV infecting humans. Insofar as inputs to the algorithm included the precise and long-standing host-specificity of EriCoV to European hedgehogs, and the absence of any record of any other coronaviruses in this species (Supplementary Data 2, 4 in ref. 1), it is not intuitive that such co-infection would have been deemed likely - the only predisposing factor being the occasional close association to humans (a factor which, if necessary, might be rather easily regulated to ensure that the risk of exposure from feeding garden hedgehogs was low). Nevertheless, it is relevant to keep in mind that the absence of evidence for SARS-CoV-2 and wildlife strains of coronaviruses other than EriCoV infecting hedgehogs does not necessarily reflect reality, as it simply may have not been detected yet, in spite of a range of studies investigating this^2–5,14^.

Anticipating the likely provenance of new strains of coronaviruses and their hosts is important research, and computer-based investigations of large datasets are an innovative approach to gaining such insight. However, this analytical power can usefully be tempered by wider biological insight, and mindful of unintended consequences. The undeniably interesting and important publication by Wardeh et al.^1^ was reported in print and electronic media nationally and globally, the vast majority of which focused primarily on hedgehogs as a previously unforeseen threat in our own backyards. A flavor of this coverage is provided, far from uniquely, by the Daily Star headline: “Bombshell study claims hedgehogs could harbor new strains of coronavirus”^15^. Numerous hedgehog rescue centres contacted us and the British Hedgehog Preservation Society, alarmed that they had received calls from members of the public asking for hedgehogs to be removed from their gardens as they could spread SARS-CoV-2.

In this wider context, remembering the exclusion of the ACE2 receptors as predictors of susceptibility to SARS-CoV-2, and the generally low interpretability of Gradient Boosting Machine (GBM) based methods, it would seem prudent to be cautious in raising public anxieties, which, to judge by the media responses in this case, can undermine public perceptions of nature, apparently with little cause. Conservation is a highly transdisciplinary subject^16^, which often necessitates tough choices attentive to social justice including in the context of epizootics. Sometimes, therefore, it is necessary that messaging about wild species is nuanced to explain both reasons to conserve and reasons to control. However, considering the fragility of public support for wildlife, it is important, although difficult, to minimize the likelihood of the media raising unwarranted fears that risk undermining conservation efforts of a species that is already in worrying decline^17^.
